# Supplementary material for: Nuclear spin diffusion in the central spin system of a GaAs/AlGaAs quantum dot
Source: Nat Commun. 2023 May 9;14:2677. doi: 10.1038/s41467-023-38349-0 (PMC10170165; doi:10.1038/s41467-023-38349-0)
Supplement: Supplementary file 1 — Supplementary Information [file 41467_2023_38349_MOESM1_ESM.pdf]

Supplementary Information: Nuclear spin diffusion in the central spin system of a GaAs/AlGaAs quantum dot

Peter Millington-Hotze and Evgeny A. Chekhovich*

Department of Physics and Astronomy, University of Sheffield, Sheffield S3 7RH, United Kingdom

Santanu Manna, Saimon F. Covre da Silva, and Armando Rastelli

Institute of Semiconductor and Solid State Physics, Johannes Kepler University Linz, Altenberger Str. 69, 4040 Linz, Austria

Supplementary Note 1. SAMPLE STRUCTURE

The sample is grown using molecular beam epitaxy (MBE) on a semi-insulating GaAs (001) substrate. The growth starts with a layer of $Al_{0.95}Ga_{0.05}As$ followed by a single pair of $Al_{0.2}Ga_{0.8}As$ and $Al_{0.95}Ga_{0.05}As$ layers acting as a Bragg reflector in optical experiments. Then, a 95 nm thick layer of $Al_{0.15}Ga_{0.85}As$ is grown. The rest of the structure follows the schematic shown in Fig. 2c of the main text beginning with a 95 nm thick layer of $Al_{0.15}Ga_{0.85}As$ doped with Si at a volume concentration of 1.0×10^{18} cm^{-3}. The low Al concentration of 0.15 in the Si doped layer mitigates the issues caused by the deep DX centers [1–3]. Under optical excitation this $Al_{0.15}Ga_{0.85}As$:Si gives rise to broad photoluminescence between 730 nm and 770 nm as observed in Supplementary Fig. 1a. The n-type doped layer is followed by the electron tunnel barrier layers: first a 15 nm thick $Al_{0.15}Ga_{0.85}As$ layer and then a 15 nm thick $Al_{0.33}Ga_{0.67}As$ layer. Aluminium droplets are grown on the surface of the $Al_{0.33}Ga_{0.67}As$ layer and are used to etch the nanoholes [4, 5]. An atomic force microscopy (AFM) image of a similar sample in Fig. 2a of the main text shows a typical nanohole with a depth of ≈ 6.5 nm and ≈ 70 nm in diameter. Next, a 2.1 nm thick layer of GaAs is grown to form QDs by infilling the nanoholes as well as to form the quantum well (QW) layer. Thus, the maximum height of the QDs in the growth z direction is ≈ 9 nm. Low temperature PL of QDs and QW is observed [Supplementary Fig. 1a] at 785 nm and 690 nm, respectively. The GaAs layer is followed by a 268 nm thick $Al_{0.33}Ga_{0.67}As$ barrier layer. Finally, the p-type contact layers doped with C are grown: a 65 nm thick layer of $Al_{0.15}Ga_{0.85}As$ with a 5×10^{18} cm^{-3} doping concentration, followed by a 5 nm thick layer of $Al_{0.15}Ga_{0.85}As$ with a 9×10^{18} cm^{-3} concentration, and a 10 nm thick layer of GaAs with a 9×10^{18} cm^{-3} concentration.

* e.chekhovich@sheffield.ac.uk

The sample is processed into a $p-i-n$ diode structure. Mesa structures with a height of 250 nm are formed by etching away the p-doped layers and depositing Ni(10 nm)/AuGe(150 nm)/Ni(40 nm)/Au(100 nm) on the etched areas. The sample is then annealed to enable diffusion down to the n-doped layer to form the ohmic back contact. The top gate contact is formed by depositing Ti(15 nm)/Au(100 nm) on to the p-type surface of the mesa areas. The sample gate bias V_{Gate} is the bias of the p-type top contact with respect to the grounded n-type back contact. By changing V_{Gate} the equilibrium charge state of the quantum dot is tuned using the Coulomb blockade effect (see Supplementary Note 2 E). Due to the large thickness of the top $Al_{0.33}Ga_{0.67}As$ layer, the tunneling of the holes is effectively blocked, whereas tunnel coupling to the n-type layer enables deterministic charging of the quantum dots with electrons.

Supplementary Note 2. EXPERIMENTAL DETAILS AND ADDITIONAL RESULTS

The sample is placed in a liquid helium bath cryostat. A superconducting coil is used to apply magnetic field up to $B_{\mathrm{z}} = 10$ T. The field is parallel to the sample growth direction and the optical axis z (Faraday geometry). We use confocal microscopy configuration. An aspheric lens with a focal distance of 1.45 mm and NA=0.58 is used as an objective for optical excitation of the QD and for photoluminescence (PL) collection. The excitation laser is focused into a spot with a diameter of ≈ 1 μm. The collected PL is dispersed in a two-stage grating spectrometer, each stage with a 0.85 m focal length, and recorded with a charge-coupled device (CCD) camera. The changes in the spectral splitting of a negatively charged trion X^-, derived from the PL spectra, are used to measure the hyperfine shifts E_{hf} proportional to the nuclear spin polarization degree.

Supplementary Fig. 2 is a detailed version of Fig. 4a of the main text and shows the timing of the NSR measurement. In what follows we describe the individual elements of the timing sequence. While this discussion is specific to the NSR measurement, the same principles apply to other time-resolved measurements. The differences applicable to NMR spectroscopy and diffusion reflux measurements are highlighted below accordingly.

A. Radiofrequency depolarization of nuclear spin polarization

Investigation of spin diffusion relies on the ability to prepare a reproducible spatial distribution of the nuclear spin polarization. This is achieved with a radiofrequency (RF) erase pulse (Supplementary Fig. 2) which effectively resets the nuclear spin polarization to zero in the entire sample.

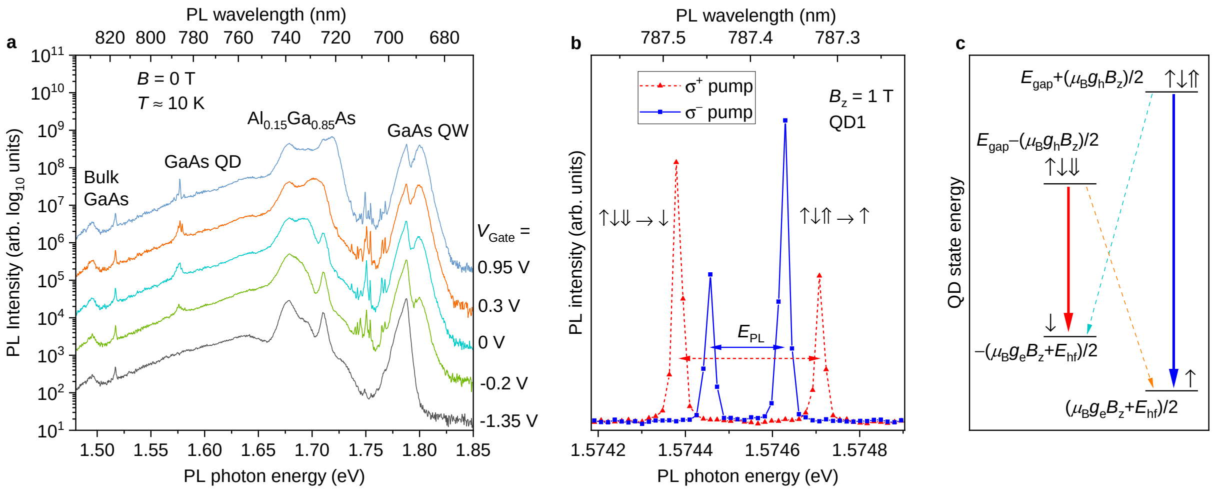

Supplementary Figure 1. **Photoluminescence of GaAs/AlGaAs QD samples.** **a** Broad range photoluminescence (PL) spectra measured under 532 nm laser excitation at different gate biases $V_{\rm Gate}$. Spectra are offset in a vertical direction (log scale) by a factor of 10 for clarity. Spectral features arising from the different parts of the sample are labeled accordingly. **b** High resolution PL spectra of a negatively charged X^- trion following σ^+ (triangles) or σ^- (squares) circularly polarized optical pumping, which creates $s_z = -1/2$ (↓) and $s_z = +1/2$ (↑) spin polarized electrons, respectively. The electrons transfer their spin to the nuclei via magnetic (hyperfine) interaction, resulting in a build up of negative or positive net nuclear spin polarization, respectively. Through the same hyperfine interaction, the average nuclear spin polarization shifts the $s_z = -1/2$ and $s_z = +1/2$ electron spin energy levels in the opposite directions. These Overhauser shifts ($E_{\rm hf}$) lead to the observed change in the spectral splitting $E_{\rm PL}$ of the trion PL, where the two components of the doublet correspond to an electron-hole recombination in presence of another electron with $s_z = -1/2$ or $s_z = +1/2$ state. **c** Energy level diagram. The electron ground state is split by the Zeeman energy $\mu_{\rm B} g_{\rm e} B_z$ and the hyperfine shift $E_{\rm hf}$. The X^- trion energy includes the QD bandgap energy $E_{\rm gap}$ and the Zeeman splitting of the unpaired hole with a positive (⇑) or negative (⇓) momentum projection. The valence band hole hyperfine effect can be neglected due to its smaller magnitude [6]. The electron and hole g-factors are $g_{\rm e}$ and $g_{\rm h}$, respectively, with $|g_{\rm h}| \gg |g_{\rm e}|$ in the studied QDs. Solid arrows depict the two optically allowed transitions responsible for the spectral doublet in (b). The dashed lines show the two forbidden "diagonal" transitions.

This is achieved by saturating the nuclear magnetic resonance of the As and Ga isotopes. When subject to an oscillating magnetic field, resonant with the nuclear Larmor frequency, the nuclear spins undergo Rabi rotation, periodically transitioning between the spin states parallel and antiparallel to the external magnetic field [7]. Due to the nuclear-nuclear dipolar interactions each nuclear spin is subject to a local field. The randomness of these local fields perturbs the Rabi precession frequencies, resulting in ensemble dephasing. Consequently, the nuclei become randomly oriented

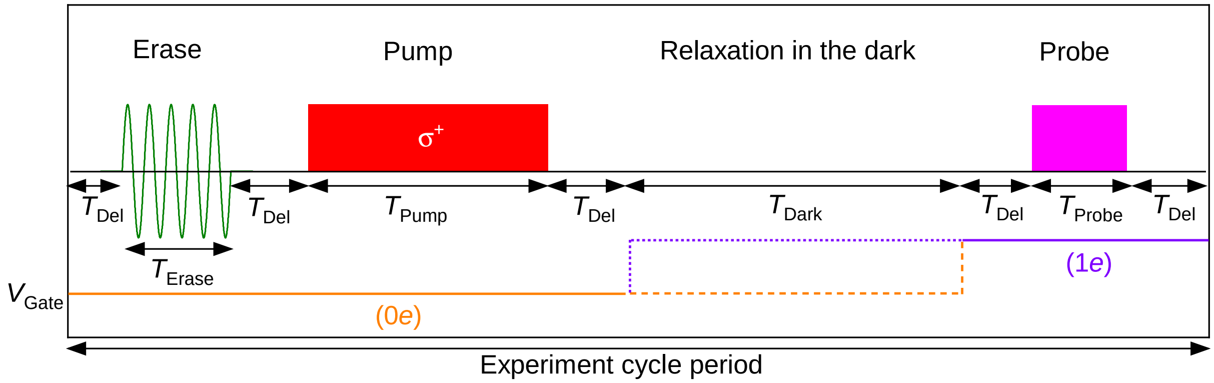

Supplementary Figure 2. **Timing diagram of the nuclear spin relaxation measurement cycle.**

(depolarized) after a long resonant radiofrequency saturation pulse. The required oscillating magnetic field $B_{\mathrm{x}} \perp z$ is produced by a coil placed at a distance of ≈ 0.5 mm from the QD sample. The coil is made of 10 turns of a 0.1 mm diameter enameled copper wire wound on a ≈ 0.4 mm diameter spool in 5 layers, with 2 turns in each layer. The coil is driven by a class-A RF amplifier (rated up to 20 W) which is fed by the output of an arbitrary waveform generator. The spectrum of the RF excitation consists of three bands, each 340 kHz wide and centered on the NMR frequency of the corresponding As or Ga isotope. For each magnetic field the frequencies are adjusted based on NMR spectroscopy. To give a specific example, the central frequencies at 10 T are 73.079, 102.471 and 130.199 MHz for ^{75}As, ^{69}Ga and ^{71}Ga, respectively. Each RF band is generated as a frequency comb [6] with a mode spacing of 120 Hz, much smaller than the homogeneous NMR linewidth. The RF power density in the comb is chosen to be low enough and the RF pulse duration T_{Erase} long enough (ranging between 0.1 and 10 s depending on magnetic field) to achieve noncoherent exponential depolarization of the nuclear spin ensemble.

B. Optical pumping of the quantum dot nuclear spins

Optical pumping of the QD nuclear spin polarization (labelled Pump in Supplementary Fig. 2) is achieved using the emission of a 690 nm circularly polarized diode laser, which is resonant with the GaAs QW states, as seen in Supplementary Fig. 1a. Optical dynamical nuclear spin polarization is a well known process, that has been observed in many types of QDs [8–12], see Ref. [13] for a review. In brief, dynamic nuclear polarization is a three-stage cyclic process. At the first stage a spin polarized electron is created optically. This is made possible by the selection rules, which allow conversion of the circularly polarized photons into spin-polarized electron-hole pairs in group

III-V semiconductors. At the second stage, the electron exchanges its spin with one of the nuclei through the flip-flop term of the electron-nuclear hyperfine Hamiltonian. The third stage is the electron-hole optical recombination, which removes the flipped electron. This final step is required in order to let the QD accept new spin-polarized electrons and continue polarizing the ensemble of $\approx 10^5$ nuclear spins of the QD. Given that optical pumping is resonant with the QW, it is possible that dynamical nuclear polarization takes place not only in the QDs but also in the adjacent parts of the QW. On the other hand, the pump laser photon energy is well below the bandgap of the AlGaAs barriers. For that reason we assume that dynamic nuclear polarization in AlGaAs is induced only through spin diffusion from the GaAs layer of the QW and QDs. During the optical pump the sample gate is set to a large reverse bias, typically $V_{\rm Gate} = -2$ V. The pump power is ≈ 300 μW, which is two orders of magnitude higher than the ground-state PL saturation power. The resulting hyperfine shifts do not exceed $|E_{\rm hf}| < 50$ μeV, corresponding to initial nuclear spin polarization degree within $|P_{\rm N,0}| \lesssim 0.4$. While polarization as high as $P_{\rm N,0} \approx 0.8$ is possible [14], we deliberately use lower values to ensure linear regime of spin diffusion, free from hyperpolarization regime corrections [15]. In the diffusion reflux experiments, presented in Fig. 3 of the main text, the first (long) pump pulse is as described above. The second (shorter) pump is chosen to have a higher power ≈ 3000 μW and a longer wavelength ≈ 793 nm, resonant with the s-shell exciton transition in order to generate inverted nuclear spin polarization localised to the QD volume.

C. Optical probing of the quantum dot nuclear spins

For optical probing of the nuclear spin polarization we use a diode laser emitting at 640 nm. Sample forward bias, typically +0.5 V, and the probe power are chosen to maximize (saturate) PL intensity of the ground state X^- trion. Supplementary Fig. 1b shows X^- PL probe spectra measured at $B_{\rm z} = 1$ T following optical pumping with σ^+ (triangles) or σ^- (squares) circular polarization. The difference in spectral splitting of the X^- trion doublet reveals the hyperfine shifts $E_{\rm hf}$ [see energy level diagram in Supplementary Fig. 1c]. These shifts are used to monitor the average QD nuclear spin polarization in NSR experiments such as shown in Fig. 4b of the main text. Illumination with a probe laser inevitably acts back on the nuclear spin polarization. An example of the probe pulse calibration is shown in Supplementary Fig. 3. In this experiment the QD is first pumped with a σ^+ or σ^- polarized laser in order to create large initial nuclear polarization. Then a probe laser pulse is applied. The hyperfine shift $E_{\rm hf}$ is measured from PL spectroscopy at the end of this probe. Such calibration is carried out for each individual QD at each

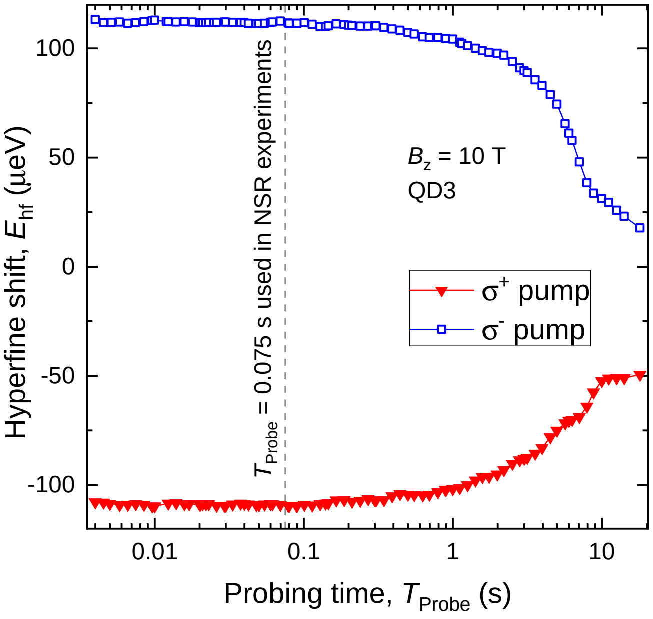

Supplementary Figure 3. **Calibration of the optical probing of the QD nuclear spin polarization.** Hyperfine shift measured as a function of the probing time $T_{\rm Probe}$ following a σ^+ or σ^- pumping of the nuclear spin polarization in a QD. Vertical dashed line shows the $T_{\rm Probe}$ value chosen for the NSR measurements on this individual QD at this particular magnetic field of $B_{\rm z} = 10$ T.

magnetic field. It can be seen that the probe induces decay of the nuclear spin polarization on a timescale of a few seconds. The probe time $T_{\rm Probe}$ used in the NSR experiments is chosen to ensure minimal distortion of the measured $E_{\rm hf}$. For example, for the data shown in Supplementary Fig. 3 we choose $T_{\rm Probe} = 0.075$ s which limits the parasitic depolarization to less than 1% of the true hyperfine shift $E_{\rm hf}$. Typical $T_{\rm Probe}$ values range between 10 and 80 ms, depending on individual QD and magnetic field.

D. Quantum dot electron g-factors

The energy splitting of the two electron spin states $\Delta E_{\rm e}$ is a sum of the Zeeman splitting $\mu_{\rm B} g_{\rm e} B_{\rm z}$ (where $\mu_{\rm B}$ is the Bohr magneton) and the hyperfine splitting $E_{\rm hf}$, arising from the nuclear spin polarization. We quantify the g-factor $g_{\rm e}$ of a resident electron using photoluminescence spectroscopy of a negatively charged trion. In Faraday geometry, two out of four optical transitions are forbidden, so that only the difference $g_{\rm h} - g_{\rm e}$ of the heavy hole and electron g-factors can be accessed. In order to derive the individual g-factors, we measure photoluminescence in oblique field configuration, where the sample growth axis is tilted by $\theta \approx 12^\circ$ away from the static magnetic

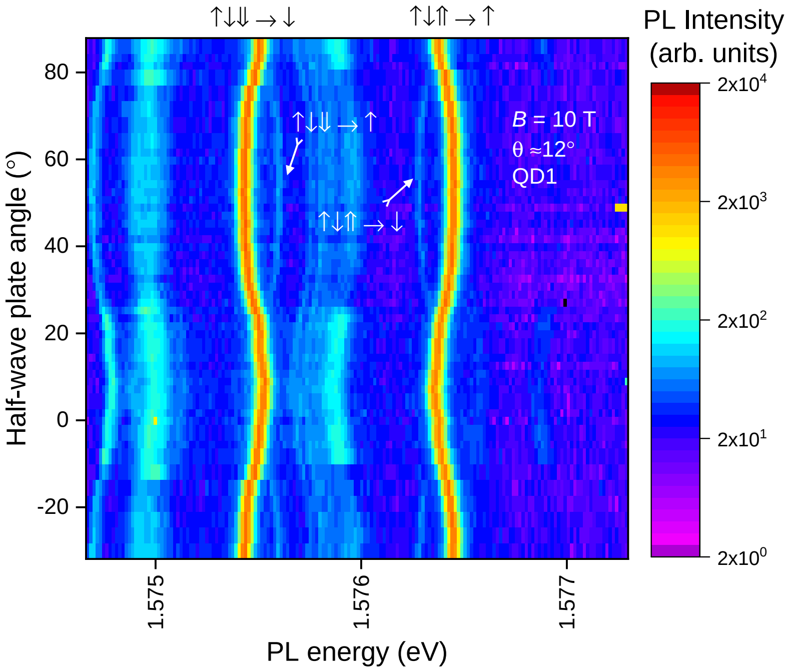

Supplementary Figure 4. **Electron g-factor measurement.** Photoluminescence spectra of a negatively charged trion X^- measured in oblique magnetic field $B = 10$ T tilted by $\theta \approx 12°$ from the Faraday geometry. The measurement uses a pump-probe protocol, where the angle of a half-wave plate on the pump laser is varied, while the probe laser is used to detect the resulting changes in PL spectrum. The two bright lines correspond to the two allowed transitions. When the circularly polarized pump generates a sufficiently large hyperfine shift, the two weakly allowed trion transitions, labeled by the arrows, become resolved. Fitting of the PL energies reveals the electron and hole g-factors. Other (broad) spectral features correspond to PL of excitons charged with more than one electron.

field. In this configuration the "diagonal" transitions, shown by the dashed lines in Supplementary Fig. 1c, become weakly allowed. Owing to the nearly vanishing electron g-factor in this type of GaAs/AlGaAs QDs [11], all four X^- transitions can be resolved in our setup only in high magnetic field $B = 10$ T and in presence of the optically induced hyperfine shifts. The experiment is conducted using an optical pump-probe method. The probe PL spectra are shown in Supplementary Fig. 4 as a function of the half-wave plate angle. The angle is varied to control the degree of circular polarization of the pump laser and the resulting hyperfine shift $E_{\rm hf}$. The two weak transitions (labeled by the arrows) become visible when the splitting of the two bright transitions is maximized by the hyperfine shift. We further measure the spectral splitting of the two bright transitions after RF depolarization of the nuclei, which results in $E_{\rm hf} \approx 0$. It is then possible to perform linear fit of the PL energies of all four X^- transitions and derive the g-factors. For QD1

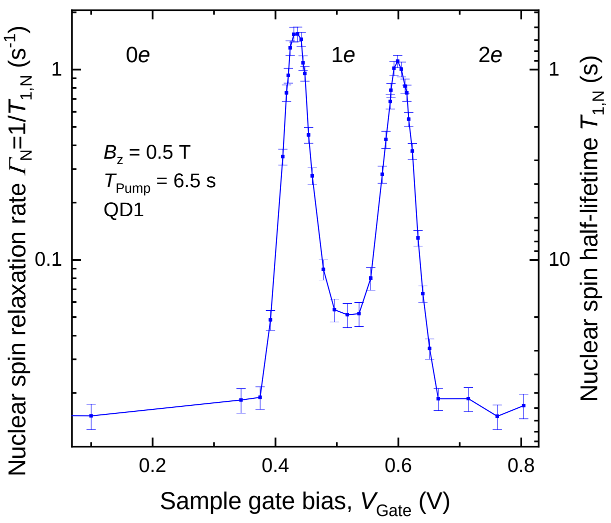

Supplementary Figure 5. **Bias dependence of the nuclear spin relaxation rate $\mathit{\Gamma}_\mathrm{N}$.** Error bars are 95% confidence intervals.

studied in the main text and examined in Supplementary Fig. 4 we find the 95% confidence estimate $g_\mathrm{e} \approx -0.101 \pm 0.007$ for the g-factor of a single resident electron. From the hole spin splitting of X^- at $B = 10$ T we estimate the hole g-factor in presence of two electrons to be $g_\mathrm{h} \approx +1.68$. This value should be treated as a rough estimate because of the significant nonlinearity in hole Zeeman splitting for this type of QDs [11]. We also measure the g-factors in a neutral exciton X^0, using PL of the dark states: we find $g_\mathrm{e} \approx -0.090 \pm 0.035$ for the electron in presence of one hole. It is notable that the electron g-factor is nearly unaffected by the extra hole [16]. Using PL spectroscopy of the X^- trion state, we have measured g-factors in two more QDs from the same sample to find $g_\mathrm{e} \approx -0.077 \pm 0.018$ and $g_\mathrm{e} \approx -0.107 \pm 0.002$ for a sole resident electron in QD6 and QD7, respectively. From the X^0 PL of QD6 we find $g_\mathrm{e} \approx -0.12 \pm 0.01$ for an electron in presence of a hole, whereas no dark excitons could be observed in QD7. The g-factors found here are in good agreement with the previous studies on the samples where QDs were grown in nanoholes etched in pure GaAs [11].

E. Quantum dot charge state tuning

The sample gate bias V_Gate is controlled by the output of an arbitrary waveform generator connected through a 1.9 MHz low pass filter. During the dark evolution time T_Dark the bias can be set to an arbitrary value. For an empty dot regime ($0e$) we use large reverse bias $V_\mathrm{Gate} = -1.3$ V. The bias corresponding to $1e$ Coulomb blockade is found by measuring the bias dependence of

$\mathit{\Gamma}_{\mathrm{N}}(V_{\mathrm{Gate}})$ such as shown in Supplementary Fig. 5. In agreement with the previous studies on InGaAs QDs [17, 18] we observe tunnelling peaks (at ≈ 0.43 V and ≈ 0.6 V), where the electron Fermi reservoir energy matches the QD charging energy. Under these resonant conditions NSR is accelerated by a non-diffusion mechanism, where the nuclear spin momentum is carried into the Fermi reservoir by the rapidly cotunnelling electron. A bias at the middle of the Coulomb valley between the peaks, 0.517 V in this case, is used to charge the QD with one electron ($1e$). Supplementary Fig. 5 shows that when the QD is charged with two electrons ($2e$) forming a spin singlet, the NSR rate is identical to the $0e$ case, confirming that the NSR acceleration produced by the single electron ($1e$) is related to its spin.

F. Pump probe experiment implementation

Optical pump and probe pulses are formed by mechanical shutters with a switching time of a few milliseconds. In order to accommodate these shutter transients, small delays $T_{\mathrm{Del}} = 10$ ms are introduced in the timing sequences as shown in Supplementary Fig. 2. Under certain regimes in B_{z} and V_{Gate} (e.g. resonant cotunnelling with the Fermi reservoir) this T_{Del} is comparable to the nuclear spin relaxation times $T_{1,\mathrm{N}}$. However, the relaxation time in an empty ($0e$) or singly charged ($1e$) QD is always considerably longer. Thus, during the switching delay the QD is kept under either the $0e$ bias (after the pump) or the $1e$ bias (prior to the probe). The dark time T_{Dark} is implemented by pulsing the gate bias to the chosen dark-state value V_{Gate} for a duration T_{Dark}. The QD device responds to the bias on a sub-microsecond scale. This way we ensure that the switching delays T_{Del} have minimal effect on the measured NSR dynamics.

G. Nuclear magnetic resonance of individual quantum dots

Nuclear magnetic resonance (NMR) characterization (Fig. 2e of the main text) is conducted using the inverse NMR method [19] which enhances the signal for $I > 1/2$ spins and improves the signal to noise ratio. In this method the nuclear spins are first polarised with a pump laser ($T_{\mathrm{Pump}} = 6.5$ s) and are then depolarized by a weak RF field, whose spectral profile is a broadband frequency comb with a narrow gap of width w_{gap} in the center. The frequency comb has a total spectral width of 600 kHz and its mode spacing is 125 Hz. The value of w_{gap} controls the balance between the measured NMR signal and the spectral resolution. In an empty QD ($0e$), NMR spectra of As and Ga measured with $w_{\mathrm{gap}} = 6$ kHz consist of well-resolved quadrupolar-split

triplets, consistent with previous observations for similar QD structures [11, 20]. The spin of a single electron ($1e$) leads to inhomogeneous Knight shifts comparable to the quadrupolar splitting. As a result, the NMR triplets are no longer resolved (solid circles in Fig. 2e of the main text). Moreover, the electron spin lifetime, which is on the order of milliseconds in the studied QDs, is much shorter than the radiofrequency burst (typically 0.18 s), and the average electron spin polarization is therefore close to 0. Each nucleus then experiences both positive and negative Knight shifts during the RF burst. These dynamic spectral shifts disrupt the enhancement of the inverse NMR method: for example, if a nuclear spin transition fits into the RF spectral gap $w_{\rm gap}$ under one sign of the Knight shift, it may be moved out of the gap and into resonance with the depolarizing RF field under the opposite Knight shift. As a result, the NMR spectrum amplitude is reduced in the $1e$ measurement. By varying the gap width $w_{\rm gap}$, we find that a spectrum with a reasonable signal to noise ratio is obtained at $w_{\rm gap} = 70$ kHz, as shown by the circles in Fig. 2e of the main text. Although the deterioration of the inverse NMR method precludes an accurate measurement of the NMR lineshape in presence of the electron, the overall width ≈ 50 kHz of the resonance still provides a valid order-of-magnitude estimate of the Knight shifts experienced by the nuclear spins in the QD. More sophisticated measurements, using pulsed NMR (to be reported separately elsewhere) confirm this rough estimate based on inverse NMR measurement.

H. Additional data on nuclear spin relaxation

The QD NSR curves measured at $B_{\rm z} = 0.39$ T and shown in Fig. 4b of the main text are reproduced in Supplementary Fig. 6a, together with the similar measurements carried out at high magnetic field $B_{\rm z} = 9.82$ T and shown in Supplementary Fig. 6b. Similar to the low fields, at $B_{\rm z} = 9.82$ T shorter optical pumping time $T_{\rm Pump}$ results in faster NSR through spin diffusion. However, the acceleration of NSR in presence of a single electron ($1e$) is less pronounced at high magnetic field, owing to the reduction of the hyperfine-mediated nuclear-nuclear spin interaction. It is also worth noting that the optical spin pumping becomes slower at high magnetic field. While $T_{\rm Pump} = 0.018$ s at $B_{\rm z} = 0.39$ T is sufficient to achieve $\approx 1/4$ of the steady state nuclear spin polarization, it takes an order of magnitude longer $T_{\rm Pump} = 0.17$ s to reach the same $\approx 1/4$ level at $B_{\rm z} = 9.82$ T. This difference limits the shortest $T_{\rm Pump}$ for which NSR dynamics can be measured at high magnetic field, as can be seen in Fig. 4d of the main text.

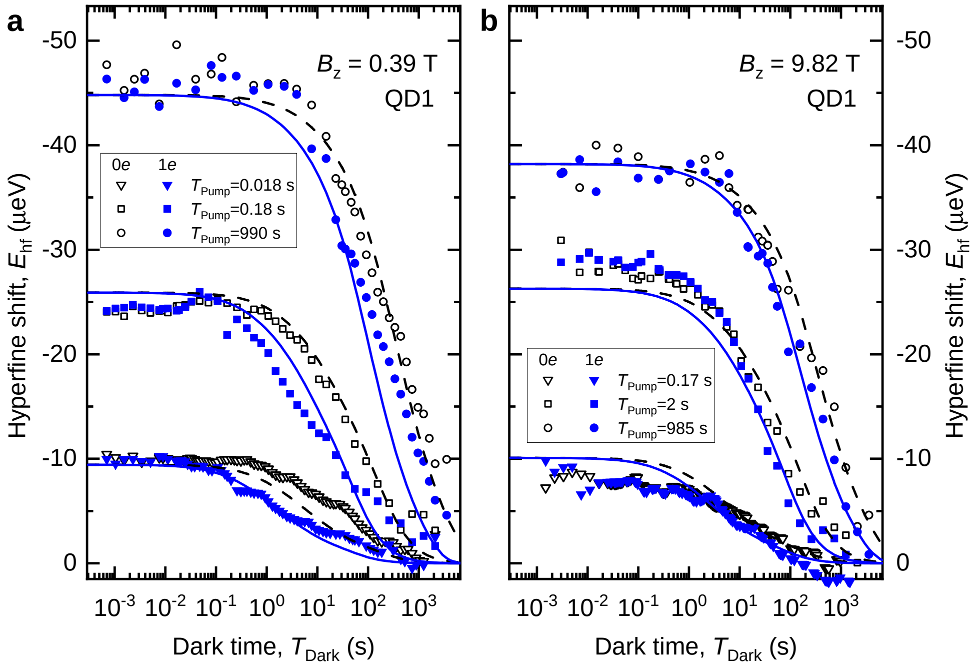

Supplementary Figure 6. **Nuclear spin relaxation in GaAs quantum dots. a** Dark time dependence of the hyperfine shift E_{hf}, which probes average nuclear spin polarization weighted by the QD electron density $|\psi_{\mathrm{e}}|^2$. Nuclear spin decay is measured (symbols) at $B_z = 0.39$ T for different pumping times T_{Pump} while keeping QD empty ($0e$, open symbols) or electron-charged ($1e$, solid symbols) during the dark time. Lines show numerical solution of the spin diffusion equation. **b** Same as (a) but for $B_z = 9.82$ T.

I. Nuclear spin relaxation at elevated temperatures

The experiments presented in the main text are conducted at the cryostat base temperature, measured with a resistive sensor to be $T \approx 4.27$ K. Additional measurements, similar to those shown in Fig. 4c,d of the main text, have been conducted on an empty QD ($0e$) at an elevated temperature $T = 15.2$ K and are shown in Supplementary Fig. 7. We find that at high temperature the relaxation rate follows the same trend of reduction at short pumping times T_{Pump}, consistent with NSR dominated by spin diffusion. In case of a pure spin diffusion driven by nuclear dipole-dipole interactions, one would expect the rate to be independent of the temperature. From Supplementary Fig. 7 we find that for any given T_{Pump} the relaxation is slightly accelerated at $T = 15.2$ K. One possibility is that temperature dependence of the optical nuclear spin pumping process [21] creates different spatial distributions of the nuclear spin polarization for the same T_{Pump}. Contribution of the temperature-dependent non-diffusion mechanisms, such as two-phonon quadrupolar relaxation is also possible, but expected to be small below 20 K (Refs. [22, 23]), in agreement with Fig. 7.

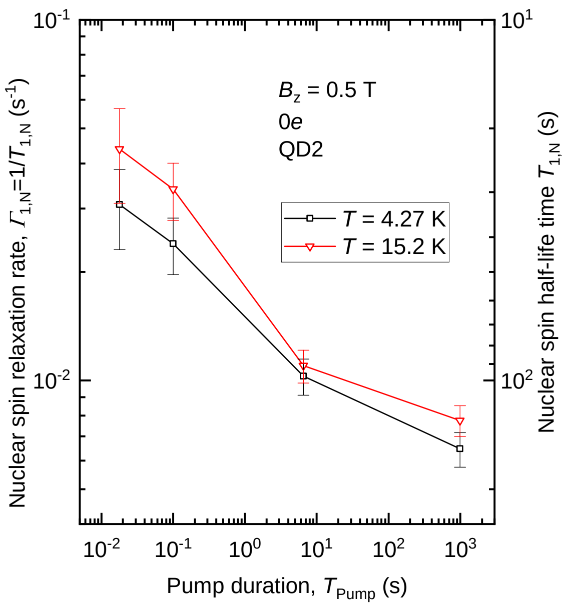

Supplementary Figure 7. **Temperature dependence of the quantum dot nuclear spin relaxation.** Fitted QD nuclear spin half-life times $T_{1,\mathrm{N}}$ (right scale) and the corresponding NSR rates $\mathit{\Gamma}_{\mathrm{N}} = 1/T_{1,\mathrm{N}}$ (left scale) measured for different pumping times T_{Pump} at $B_{\mathrm{z}} = 0.5$ T. Experiments are conducted at base sample temperature ($T = 4.27$ K, squares) and an elevated temperature ($T = 15.2$ K, triangles). Error bars are 95% confidence intervals.

Supplementary Note 3. FIRST PRINCIPLE ESTIMATE OF THE GaAs NUCLEAR SPIN DIFFUSION COEFFICIENT

In the absence of free electrons, nuclear spin diffusion is driven by the dipole-dipole magnetic nuclear spin interaction. The total dipole-dipole Hamiltonian term is a sum of pairwise couplings:

$$\mathcal{H}_{\mathrm{DD}} = \sum_{i<j} b_{i,j}\left(2\hat{I}_{\mathrm{z},i}\hat{I}_{\mathrm{z},j} - \hat{I}_{\mathrm{x},i}\hat{I}_{\mathrm{x},j} - \hat{I}_{\mathrm{y},i}\hat{I}_{\mathrm{y},j}\right),$$
$$b_{i,j} = \frac{\mu_0\hbar^2}{4\pi}\frac{\gamma_i\gamma_j}{2}\frac{1-3\cos^2\theta_{i,j}}{r_{i,j}^3}, \tag{S1}$$

where $\mu_0 = 4\pi\times10^{-7}$ NA^{-2} is the magnetic constant, $\hbar$ is the reduced Planck's constant and $r_{i,j}$ denotes the length of the vector, which forms an angle θ with the static magnetic field direction (z) and connects the two spins i and j. The typical magnitude of the interaction constants for the nearby nuclei in GaAs is $\max(|b_{j,k}|)/h \approx 100$ Hz. The Hamiltonian of Eq. S1 has been truncated to eliminate all spin non-conserving terms, which is justified for static magnetic fields exceeding $\gtrsim 1$ mT, as used in this work. The evolution of a large nuclear spin ensemble can be described in terms of spin diffusion with coefficient D. In crystalline solids the nuclear spin diffusion

coefficient D, is a rank-2 tensor which can be calculated from the first principles using density matrix approach [24] or the method of moments [25, 26]. The calculation involves a somewhat lengthy evaluation of the various lattice sums. Here we use a more recent version of the method of moments from Ref. [27]. We re-evaluate numerically the sums of Eqns. 8 and 10 from Ref. [27] using an FCC lattice of 6859 spins. Our results are in good agreement with the those derived for 1330 neighboring spins previously [27]. We find the following values for the diagonal components of D: $D_{xx} = D_{yy} \approx 0.2594 \frac{\mu_0}{4\pi} \frac{\hbar\gamma^2}{a_0} \rho^{1/3}$ and $D_{zz} \approx 0.3289 \frac{\mu_0}{4\pi} \frac{\hbar\gamma^2}{a_0} \rho^{1/3}$, where $a_0 \approx 0.565$ nm is the GaAs lattice constant and γ is the nuclear gyromagnetic ratio. Here we use the coordinate system aligned with the cubic crystal axes $x \parallel [100]$, $y \parallel [010]$, $z \parallel [001]$, and the strong magnetic field is parallel to the z direction. We have also introduced the correction factor $\rho^{1/3}$ to account for the increase of the average internuclear distance for the isotope whose abundance ρ is less then unity.

In case of arsenic, ^{75}As is the only stable isotope, so that $\rho = 1$. For gallium isotopes we have the natural abundances $\rho = 0.601$ and $\rho = 0.399$ for ^{69}Ga and ^{71}Ga, respectively. The gyromagnetic ratios γ are known [28] and, since we approximate the spin diffusion as a one-dimensional process along the sample growth direction z, we are interested in the D_{zz} component of the tensor. Substituting the numerical values we find $D_{zz} \approx 13, 21, 30$ nm^2 s^{-1} for ^{75}As, ^{69}Ga and ^{71}Ga, respectively. The experiments presented in this work do not resolve between spin diffusion of the individual isotopes. As a simple approximation we can treat the observed NSR dynamics as a result of spin diffusion within one type of nuclei but with a weighted average diffusion constant. We use as weights the relative contributions of the isotopes to the optically measured hyperfine shift E_{hf}. From the previous studies of the similar QDs [14] these contributions are estimated as 0.49, 0.28 and 0.23 for ^{75}As, ^{69}Ga and ^{71}Ga, respectively, from where the average diffusion coefficient is approximated as $D_{zz} \approx 19$ nm^2.

Supplementary Note 4. NUMERICAL SIMULATION OF NUCLEAR SPIN DIFFUSION

The spatiotemporal evolution of the nuclear spin polarization degree $P_{\mathrm{N}}(t, z)$ is modeled by solving the partial differential spin diffusion equation

$$\frac{\partial P_{\mathrm{N}}(t,z)}{\partial t} = D(t) \frac{\partial^2 P_{\mathrm{N}}(t,z)}{\partial z^2} + w(t)|\psi_{\mathrm{e}}(z)|^2 (P_{\mathrm{N},0} - P_{\mathrm{N}}(t,z)), \tag{S2}$$

where the last term describes optical nuclear spin pumping with a rate proportional to electron density $|\psi_{\mathrm{e}}(z)|^2$ and the time-dependent factor $w(t)$ equal to 0 or w_0 when optical pumping is off or on, respectively. The spin diffusion coefficient $D(t)$ also takes two discrete values D_{Dark} or D_{Pump}

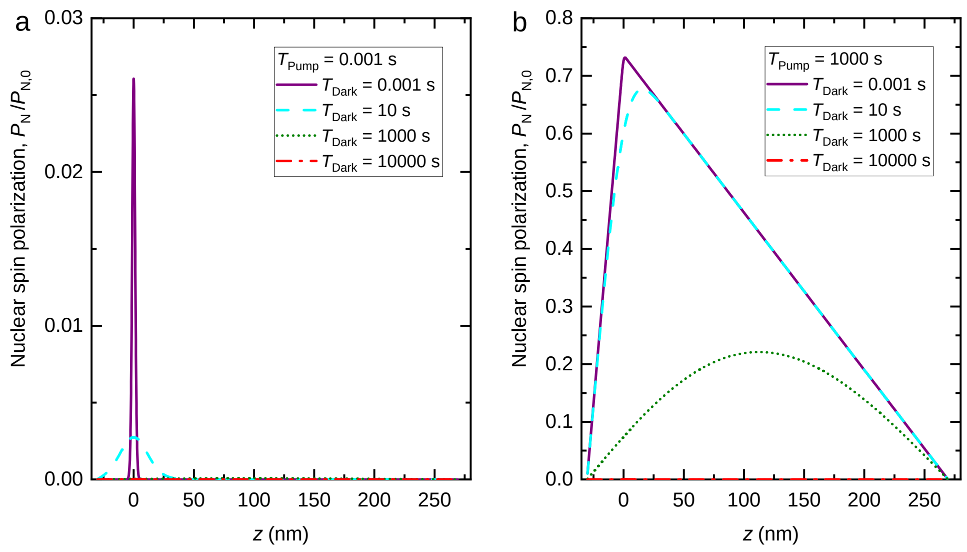

Supplementary Figure 8. **Numerical modeling of the nuclear spin diffusion.** Calculated normalized nuclear spin polarization as a function of the z coordinate at different T_{Dark}. **a** Calculations for $T_{\text{Pump}} = 1$ ms. **b** Same calculations as (a) but for $T_{\text{Pump}} = 1000$ s.

when optical pumping is off or on, respectively. $P_{\text{N},0}$ is a steady state nuclear spin polarization degree that optical pumping would generate in the absence of spin diffusion. At each time point we assume the same diffusion coefficient D across the entire structure. The equation describes a one-dimensional problem where diffusion can take place only along the z coordinate so that the nuclear spin polarization degree P_{N} does not depend on x or y. The GaAs QD layer is modeled by taking a Gaussian profile for the electron density $|\psi_{\text{e}}(z)|^2 \propto 2^{-\left(\frac{z-z_0}{h_{\text{QD}}/2}\right)^2}$, where h_{QD} is the full width at half maximum of the $|\psi_{\text{e}}(z)|^2$ function and the center of the QD is set to be $z_0 = 0$. We use Dirichlet boundary condition $P_{\text{N}} = 0$ to model fast nuclear spin depolarization in presence of the free carriers both in the n- and p-type doped layers. The boundary coordinates, where Dirichlet conditions are enforced, are chosen to match the actual sample structure as described in Supplementary Note 1. We note that the hyperfine interaction of the valence band holes is approximately 10 times weaker than for the conduction band electrons [6]. Moreover, the p-type layer is approximately 10 times further away from the QDs than the n-type layer. As a result the dynamics of the nuclear spin polarization at the QD are dominated by the n-type layer, while the exact boundary condition at the p-type layer is less important, justifying the use of the Dirichlet condition at both doped layers.

Supplementary Eq. S2 is solved numerically using the method of lines implemented in WOLFRAM MATHEMATICA 12.0. The initial condition is taken to be $P_{\text{N}} = 0 \ \forall \ z$, which models the result of

the RF Erase pulse at the start of each measurement cycle. Optical nuclear spin pumping starts at $t = -T_{\rm Pump}$ and the equation is solved until $t = 0$ with $D = D_{\rm Pump}$ and $w = w_0$. At $t = 0$ optical pumping is switched off by setting $w = 0$ and the equation is solved until $t = T_{\rm Dark}$ with $D = D_{\rm Dark}$. Supplementary Fig. 8 shows the calculated spatial profiles of the final nuclear spin polarization $P_{\rm N}(T_{\rm Dark}, z)$ normalized by its steady-state value $P_{\rm N,0}$. The results are shown for several $T_{\rm Dark}$ values in case of a short pumping (a, $T_{\rm Pump} = 1$ ms) and long pumping (b, $T_{\rm Pump} = 1000$ s). Short pumping results in a small-magnitude ($P_{\rm N} \ll P_{\rm N,0}$) spatially-narrow nuclear spin polarization, which quickly dissipates at $t > 0$. By contrast, long pumping leads to a steady-state spatial distribution where polarization peaks at the quantum dot coordinate $z = 0$ and reduces linearly towards the doped layers which act as nuclear spin polarization sinks. Interestingly, this calculation predicts that the maximum polarization $P_{\rm N,0}$ is not achieved because of the diffusion towards the doped layers, especially the closely located n-type layer at $z < 0$.

In order to compare simulations with the experimental results the final spatial distribution $P_{\rm N}(T_{\rm Dark}, z)$ is multiplied by $|\psi_{\rm e}(z)|^2$ and integrated over z. This way we reproduce the optical probing of the nuclear spin polarization, where the measured hyperfine shift $E_{\rm hf}$ is effectively weighted by the electron envelope wavefunction density $|\psi_{\rm e}(z)|^2$. The simulated hyperfine shift is then derived as $E_{\rm hf} = AIP_{\rm N}$, where I is the nuclear spin number and A is the hyperfine constant. We then use a differential evolution algorithm to vary the parameters such as $D^{(ne)}_{\rm Dark}(B_{\rm z})$, $w_0(B_{\rm z})$ and $D_{\rm Pump}(B_{\rm z})$ and fit the simulated $E_{\rm hf}$ dynamics to the entire experimental datasets of $E_{\rm hf}(T_{\rm Pump}, T_{\rm Dark})$ measured at $B_{\rm z} = 0.39$ and 9.82 T for empty ($n = 0$) and charged ($n = 1$) QD states. As discussed in the main text the best-fit diffusion coefficients in the dark are $D^{(1e)}_{\rm Dark}(9.82\ {\rm T}) = 4.7^{+1.2}_{-1.0}$ nm^2 s^{-1}, $D^{(1e)}_{\rm Dark}(0.39\ {\rm T}) = 7.7 \pm 1.9$ nm^2 s^{-1} and $D^{(0e)}_{\rm Dark} = 2.2^{+0.7}_{-0.5}$ nm^2 s^{-1} independent of magnetic field. For spin diffusion coefficients under optical pumping we find significantly larger values $D^{(1e)}_{\rm Pump}(9.82\ {\rm T}) = 96^{+44}_{-28}$ nm^2 s^{-1} and $D^{(1e)}_{\rm Pump}(0.39\ {\rm T}) = 850^{+240}_{-220}$ nm^2 s^{-1}. Such increase in D can be ascribed to the spectrally broad fluctuations of the optically generated electron spins which facilitate coupling between the distant nuclear spins, thus accelerating the spin diffusion. This is also consistent with the proposed influence of the phonon-induced electron spin flips on nuclear spin diffusion in the dark (see main text). The other best-fit parameters are $w_0(0.39\ {\rm T}) = 37^{+7}_{-5}$ s^{-1}, $w_0(9.82\ {\rm T}) = 5.7^{+1.1}_{-0.9}$ s^{-1} and $h_{\rm QD} = 2.1^{+0.3}_{-0.2}$ nm. Previous studies on GaAs/AlGaAs QDs emitting at a similar wavelength estimated that 0.92 of the electron density resides in the GaAs layer [14], whose full width can then be estimated as $h_{\rm QD}\frac{{\rm erf}^{-1}(0.92)}{\sqrt{\ln(2)}} = 3.2^{+0.4}_{-0.3}$ nm. This best-fit value somewhat underestimates the true QD thickness in z direction, but is within the range bounded by the QW thickness (2.1 nm) and the maximum QD thickness (≈ 9 nm)

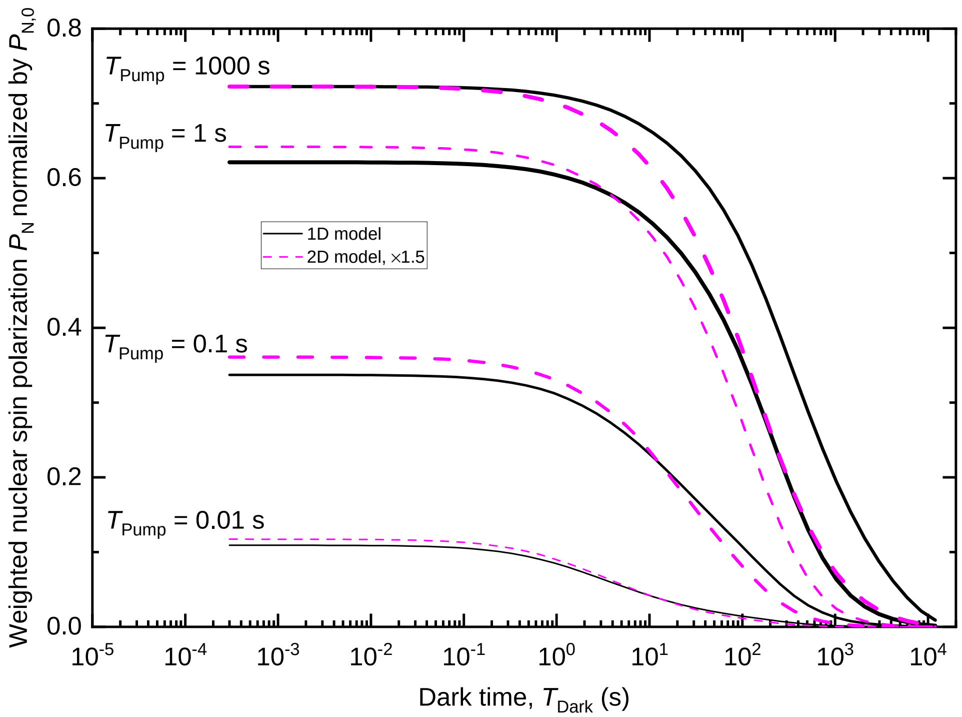

Supplementary Figure 9. **Numerical modeling of 1D and 2D nuclear spin diffusion.** Numerically simulated nuclear spin polarization degree P_{N} weighted by the electron envelope wavefunction density $|\psi_{\mathrm{e}}(z)|^2$ and normalized by the maximum nuclear spin polarization $P_{\mathrm{N},0}$ in the absence of spin diffusion. The weighted polarization is plotted as a function of the dark time T_{Dark} for different T_{Pump}. The results are shown for the case of one dimensional diffusion (1D, solid lines) and two dimensional diffusion (2D, dashed lines, P_{N} values multiplied by 1.5).

estimated from the nanohole depth in AFM. The spatial profiles shown in Supplementary Fig. 8 are calculated with the best-fit parameters for the 1e case at $B_{\mathrm{z}} = 0.39$ T.

The use of the one dimensional spin diffusion model is motivated by the large aspect ratio of the QD. Indeed, the diffusion proceeds predominantly along the direction of the strongest gradient in the nuclear spin polarization degree, which is the growth z direction. For numerical simulations, the one dimensional model is also advantageous as it requires significantly less computational resources than a full three dimensional diffusion model. In order to evaluate the limitations of the one dimensional model we run a simulation of a two dimensional diffusion problem, where the equation now reads:

$$\frac{\partial P_{\mathrm{N}}(t,x,z)}{\partial t} = D(t)\left(\frac{\partial^2 P_{\mathrm{N}}(t,x,z)}{\partial x^2} + \frac{\partial^2 P_{\mathrm{N}}(t,x,z)}{\partial z^2}\right) + w(t)|\psi_{\mathrm{e}}(x,z)|^2(P_{\mathrm{N},0} - P_{\mathrm{N}}(t,x,z)) \quad \text{(S3)}$$

The electron density is taken to be $|\psi_{\mathrm{e}}(x,z)|^2 \propto 2^{-\left(\frac{x-x_0}{d_{\mathrm{QD}}/2}+\frac{z-z_0}{h_{\mathrm{QD}}/2}\right)^2}$, where d_{QD} is a full width at

half maximum diameter of the QD, which we set to $d_{\mathrm{QD}} = 47$ nm in order to match the 0.92 electron wavefunction density in a QD with a full diameter of 70 nm. The same $|\psi_{\mathrm{e}}(x,z)|^2$ is used to calculate the weighted nuclear spin polarization degree, emulating the optical probing of the QD hyperfine shift E_{hf}. The computational domain is limited to $|x| < 700$ nm and we implement the additional Dirichlet boundary condition $P_{\mathrm{N}}(x = \pm 700\ \mathrm{nm}) = 0$.

Supplementary Fig. 9 shows the simulated QD NSR dynamics in the one dimensional (1D, solid lines) and two dimensional (2D, dashed lines) cases, following nuclear spin pumping with different durations T_{Pump}. One apparent difference in the resulting dynamics is the lower weighted nuclear spin polarization degree within the QD volume in the 2D case. Consequently, all the 2D-case P_{N} values in Supplementary Fig. 9 have been multiplied by 1.5, to simplify comparison with the 1D case. At short $T_{\mathrm{Pump}} \leq 0.1$ s QD nuclear spin polarization decays on the same timescale both in the 1D and 2D cases. This is expected since the spatial profile of the nuclear spin polarization produced by short pumping is proportional to $\propto |\psi_{\mathrm{e}}(x,z)|^2$ in the 2D case. As a result, the subsequent diffusion in the dark proceeds predominantly along the direction of the highest gradient (the growth z direction), making diffusion essentially one dimensional. By contrast, long pumping $T_{\mathrm{Pump}} \geq 0.1$ s in a 2D model makes the polarization profile more isotropic in the xz plane (for an unbounded problem at $T_{\mathrm{Pump}} \to \infty$ the polarization will tend to a profile with circular contour lines in the xz plane). In other words, after long pumping the system "forgets" the initial profile $\propto |\psi_{\mathrm{e}}(x,z)|^2$ of the QD pumping source. The subsequent diffusion in the absence of pumping (i.e. in the dark) is controlled by the dimensionality of the unpolarized space, and is seen to be faster in the 2D case. From these additional results we conclude that the one dimensional model is sufficient to capture the key aspects of QD NSR dynamics, such as slower relaxation following long optical nuclear spin pumping. However, some deviation of the 1D model from the real dynamics is inevitable, especially at long T_{Pump}, where dimensionality affects the diffusion dynamics. Such discrepancies are likely to introduce systematic errors in the best fit dynamics (Supplementary Fig. 6) and spin diffusion coefficient D values. On the other hand, in a real QD system D is not constant, and the approximate nature of the spin diffusion concept itself entails a range of systematic errors. This justifies the use of a simplified one dimensional model to describe our experimental results.

Supplementary Note 5. ELECTRON SPIN RELAXATION TIME

Here we present electron spin lifetimes $T_{1,\mathrm{e}}$ obtained from preliminary measurements in a sep-

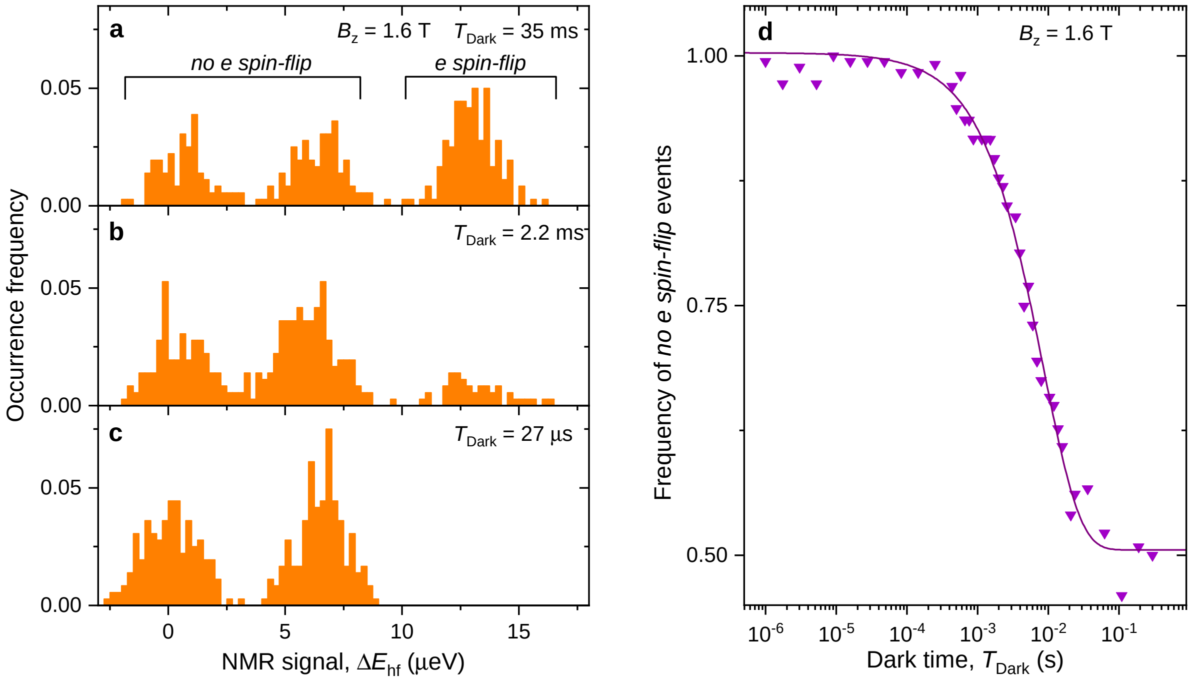

Supplementary Figure 10. **Electron spin lifetime measurements. a-c** Histograms of the single-shot NMR measurements in presence of an electron conducted for different intervals $T_{\rm Dark}$ between the two detuned RF pulses. **d** Frequency of the events where the electron spin does not flip over the $T_{\rm Dark}$ time interval (symbols). Lines shows fitting used to derive the electron spin relaxation time.

arate piece of the same epitaxial GaAs/AlGaAs QD structure. Experimental investigation of the relaxation dynamics requires a tool to measure the state of the resident electron spin trapped in a QD. We use a variation of the method demonstrated recently in Stranski-Krastanov QDs [29]. The technique uses the nuclear spin ensemble of a QD and the Knight shifts, which reduce (increase) the NMR frequency if the electron spin is in the $s_{\rm z} = -1/2$ ($s_{\rm z} = +1/2$) state. The state of the electron is encoded into the nuclear spin polarization by applying an RF pulse whose frequency is tuned to be resonant with the nuclei only if the electron is in the $s_{\rm z} = -1/2$ spin state. The amplitude and the duration of the pulse are calibrated to produce a π rotation (polarization inversion) of the nuclei when $s_{\rm z} = -1/2$. By contrast, for $s_{\rm z} = +1/2$, the Knight field detunes the nuclei out of resonance with the RF pulse, meaning that nuclear polarization is not inverted. Following the RF π pulse an optical probe is used to measure the changes in the nuclear spin polarization (the changes in the hyperfine shift $E_{\rm hf}$). In this way, a single-shot readout of the electron spin is performed with high fidelity, exceeding 99%.

We take this method one step further by applying two RF π pulses separated by a free evolution time $T_{\rm Dark}$. The first pulse performs heralded initialization of the electron spin state through

measurement, while the second RF pulse probes whether the state of the electron spin has changed during the free evolution time $T_{\rm Dark}$. There are three possible outcomes of such an experiment: (i) The electron is in the $s_{\rm z} = +1/2$ state initially and remains in this state. Both RF pulses are then out of resonance with the nuclei, so that a minimal NMR signal (variation in $E_{\rm hf}$) is expected. (ii) The electron is in the $s_{\rm z} = -1/2$ state initially and remains in this state. Both RF pulses are in resonance, so that the nuclei get rotated twice. While a 2π rotation is supposed to return the nuclei into the original state, the Knight shift inhomogeneity means that the rotations are imperfect. Thus a small but finite variation in $E_{\rm hf}$ is expected. (iii) The electron is in the $s_{\rm z} = \pm 1/2$ state initially, but flips into the opposite state $s_{\rm z} = \mp 1/2$ during the $T_{\rm Dark}$ interval. In this case one of the RF pulses will be in resonance and one out of resonance. The overall rotation will be π, so that a large change in $E_{\rm hf}$ is expected.

The results of the single-shot NMR measurements are shown in Supplementary Figs. 10a-c for different $T_{\rm Dark}$. At short $T_{\rm Dark}$ there is a bimodal distribution of the NMR signals. The two modes correspond to the no-spin-flip cases (i) and (ii), where the electron preserves its spin during $T_{\rm Dark}$. As the free evolution time becomes longer, the third mode emerges, corresponding to the spin-flip case (iii), where the electron has the opposite spin projections at the start and the end of the $T_{\rm Dark}$ interval. From such histograms, we evaluate the frequency of the events where the electron spin is not flipped and plot it as a function of $T_{\rm Dark}$ (symbols in Supplementary Fig. 10d). At short $T_{\rm Dark}$ the frequency is close to unity, while in the limit of long $T_{\rm Dark}$ the frequency tends to 0.5 because the initial and the final electron spin states become completely uncorrelated. The solid line in Supplementary Fig. 10d shows the best stretched-exponential fit, which reveals the electron spin relaxation time $T_{1,\rm e} \approx 8.5$ ms in this particular experiment conducted at $B_{\rm z} = 1.6$ T. Similar measurements at different magnetic fields yield $T_{1,\rm e} \approx 7$ ms at $B_{\rm z} = 2$ T, reducing to $T_{1,\rm e} \approx 0.5$ ms at $B_{\rm z} = 7$ T. Further details and results are to be reported by H. E. Dyte, G. Gillard, *et al.* in a forthcoming publication.

Supplementary Note 6. RAW DATA

The raw data of Fig. 3b from the main text (diffusion reflux measurement) can be found in Supplementary file Fig3b.xls. This experimental dataset is not filtered.

The raw data for nuclear spin relaxation measurements can be found in Supplementary File FigS6.xls. This file contains a full dataset, part of which is shown by the symbols in Supplementary Fig. 6 and Fig. 4b of the main text. The data shown in the figures is mildly filtered (Gaussian

kernel filter with full width at half maximum of 0.2 in $\log_{10}$ units). The filtered data is given in the first four sheets of the file. The same unfiltered data is given in the last four sheets of the file. Each sheet corresponds to a certain QD charge state and magnetic field. In each sheet, the first column is the dark time T_{Dark} (horizontal axis) and the remaining columns are the E_{hf} values (vertical axis) for different pump times T_{Pump}.

---

[1] A. Oshiyama and S. Ohnishi, DX center: Crossover of deep and shallow states in Si-doped $\mathrm{Al_xGa_{1-x}As}$, Phys. Rev. B **33**, 4320 (1986).

[2] P. M. Mooney, Deep donor levels (DX centers) in III-V semiconductors, Journal of Applied Physics **67**, R1 (1990).

[3] L. Zhai, M. C. Löbl, G. N. Nguyen, J. Ritzmann, A. Javadi, C. Spinnler, A. D. Wieck, A. Ludwig, and R. J. Warburton, Low-noise GaAs quantum dots for quantum photonics, Nat. Commun. **11**, 4745 (2020).

[4] C. Heyn, A. Stemmann, T. Koppen, C. Strelow, T. Kipp, M. Grave, S. Mendach, and W. Hansen, Highly uniform and strain-free GaAs quantum dots fabricated by filling of self-assembled nanoholes, Appl. Phys. Lett. **94**, 183113 (2009).

[5] P. Atkinson, E. Zallo, and O. G. Schmidt, Independent wavelength and density control of uniform GaAs/AlGaAs quantum dots grown by infilling self-assembled nanoholes, J. Appl. Phys. **112**, 054303 (2012).

[6] E. A. Checkhovich, M. M. Glazov, A. B. Krysa, M. Hopkinson, P. Senellart, A. Lemaître, M. S. Skolnick, and A. I. Tartakovskii, Element-sensitive measurement of the hole-nuclear spin interaction in quantum dots, Nat. Phys. **9**, 74 (2013).

[7] F. Bloch, Nuclear induction, Phys. Rev. **70**, 460 (1946).

[8] D. Gammon, A. L. Efros, T. A. Kennedy, M. Rosen, D. S. Katzer, D. Park, S. W. Brown, V. L. Korenev, and I. A. Merkulov, Electron and nuclear spin interactions in the optical spectra of single GaAs quantum dots, Phys. Rev. Lett. **86**, 5176 (2001).

[9] B. Eble, O. Krebs, A. Lemaître, K. Kowalik, A. Kudelski, P. Voisin, B. Urbaszek, X. Marie, and T. Amand, Dynamic nuclear polarization of a single charge-tunable InAs/GaAs quantum dot, Phys. Rev. B **74**, 081306 (2006).

[10] J. Skiba-Szymanska, E. A. Chekhovich, A. E. Nikolaenko, A. I. Tartakovskii, M. N. Makhonin, I. Drouzas, M. S. Skolnick, and A. B. Krysa, Overhauser effect in individual InP/$\mathrm{Ga}_x\mathrm{In}_{1-x}$P dots, Phys. Rev. B **77**, 165338 (2008).

[11] A. Ulhaq, Q. Duan, E. Zallo, F. Ding, O. G. Schmidt, A. I. Tartakovskii, M. S. Skolnick, and E. A. Chekhovich, Vanishing electron g factor and long-lived nuclear spin polarization in weakly strained nanohole-filled GaAs/AlGaAs quantum dots, Phys. Rev. B **93**, 165306 (2016).

[12] G. Ragunathan, J. Kobak, G. Gillard, W. Pacuski, K. Sobczak, J. Borysiuk, M. S. Skolnick, and E. A. Chekhovich, Direct measurement of hyperfine shifts and radio frequency manipulation of nuclear spins in individual CdTe/ZnTe quantum dots, Phys. Rev. Lett. **122**, 096801 (2019).

[13] B. Urbaszek, X. Marie, T. Amand, O. Krebs, P. Voisin, P. Maletinsky, A. Högele, and A. Imamoglu, Nuclear spin physics in quantum dots: An optical investigation, Rev. Mod. Phys. **85**, 79 (2013).

[14] E. A. Chekhovich, A. Ulhaq, E. Zallo, F. Ding, O. G. Schmidt, and M. S. Skolnick, Measurement of the spin temperature of optically cooled nuclei and GaAs hyperfine constants in GaAs/AlGaAs quantum dots, Nature Mater. **16**, 982 (2017).

[15] Y. Wang and K. Takeda, Speedup of nuclear spin diffusion in hyperpolarized solids, New Journal of Physics **23**, 073015 (2021).

[16] D. Huber, B. U. Lehner, D. Csontosová, M. Reindl, S. Schuler, S. F. Covre da Silva, P. Klenovský, and A. Rastelli, Single-particle-picture breakdown in laterally weakly confining GaAs quantum dots, Phys. Rev. B **100**, 235425 (2019).

[17] C. Latta, A. Srivastava, and A. Imamoğlu, Hyperfine Interaction-Dominated Dynamics of Nuclear Spins in Self-Assembled InGaAs Quantum Dots, Phys. Rev. Lett. **107**, 167401 (2011).

[18] G. Gillard, I. M. Griffiths, G. Ragunathan, A. Ulhaq, C. McEwan, E. Clarke, and E. A. Chekhovich, Fundamental limits of electron and nuclear spin qubit lifetimes in an isolated self-assembled quantum dot, npj Quantum Inf. **7**, 43 (2021).

[19] E. A. Chekhovich, K. V. Kavokin, J. Puebla, A. B. Krysa, M. Hopkinson, A. D. Andreev, A. M. Sanchez, R. Beanland, M. S. Skolnick, and A. I. Tartakovskii, Structural analysis of strained quantum dots using nuclear magnetic resonance, Nature Nanotechnol. **7**, 646 (2012).

[20] E. A. Chekhovich, I. M. Griffiths, M. S. Skolnick, H. Huang, S. F. Covre da Silva, X. Yuan, and A. Rastelli, Cross calibration of deformation potentials and gradient-elastic tensors of GaAs using photoluminescence and nuclear magnetic resonance spectroscopy in GaAs/AlGaAs quantum dot structures, Phys. Rev. B **97**, 235311 (2018).

[21] B. Urbaszek, P.-F. Braun, T. Amand, O. Krebs, T. Belhadj, A. Lemaítre, P. Voisin, and X. Marie, Efficient dynamical nuclear polarization in quantum dots: Temperature dependence, Phys. Rev. B **76**, 201301 (2007).

[22] R. L. Mieher, Quadrupolar nuclear relaxation in the III-V compounds, Phys. Rev. **125**, 1537 (1962).

[23] J. A. McNeil and W. G. Clark, Nuclear quadrupolar spin-lattice relaxation in some III-V compounds, Phys. Rev. B **13**, 4705 (1976).

[24] I. J. Lowe and S. Gade, Density-matrix derivation of the spin-diffusion equation, Phys. Rev. **156**, 817 (1967).

[25] A. G. Redfield and W. N. Yu, Moment-method calculation of magnetization and interspin-energy diffusion, Phys. Rev. **169**, 443 (1968).

[26] A. G. Redfield and W. N. Yu, Moment-method calculation of magnetization and interspin-energy diffusion, Phys. Rev. **177**, 1018 (1969).

[27] E. R. Butkevich and R. K. Sabirov, On anisotropy of nuclear spin-diffusion coefficient, Physica Status Solidi (b) **146**, 683 (1988).

[28] R. K. Harris, E. D. Becker, S. M. Cabral de Menezes, R. Goodfellow, and P. Granger, NMR nomenclature: Nuclear spin properties and conventions for chemical shifts: IUPAC recommendations 2001, Solid State Nuclear Magnetic Resonance **22**, 458 (2002).

[29] G. Gillard, E. Clarke, and E. A. Chekhovich, Harnessing many-body spin environment for long coherence storage and high-fidelity single-shot qubit readout, Nature Communications **13**, 4048 (2022).
